# On the Employment of Sulphate of Zinc as an Antiseptic

**Published:** 1852-03

**Authors:** 


*On the Employment of Sulphate of Zinc as an Antiseptic.*—M. Falcony states, as the result of his experimental researches, that sulphate of zinc is not only eminently antiseptic, preserving animal substances from decay, but that it actually arrests the progress of putrefaction which has once commenced. The injection of four or five quarts of the solution of this salt in water, through the arteries, suffices for the preservation of a human body, in a state of perfect flexibility for upwards of forty days. Anatomical preparations thus made, will serve for dissection for a considerable period; the use of the solution not affecting the steel instruments employed. M. Falcony has also found, that preparations which have undergone change by maceration, resume their original character when immersed in a solution of sulphate of zinc.—*London Med. Times.*

